# Complete Urogenital and Anorectal Duplication in a Dog

**DOI:** 10.1155/2019/3696978

**Published:** 2019-03-03

**Authors:** Jonathan M. Coffman, Marina McConkey, Gary Ellison, Elizabeth Huynh, Michael J. Dark

**Affiliations:** ^1^Department of Small Animal Clinical Sciences, College of Veterinary Medicine, University of Florida, Gainesville, FL 32610, USA; ^2^Department of Clinical, Diagnostic, and Population Medicine and Emerging Pathogens Institute, University of Florida, Gainesville, FL 32610, USA

## Abstract

A 10-week-old sexually intact female golden retriever was evaluated for two functional anal openings and a bipartite vulva. Examination revealed haired skin between two separate anatomically smaller anal openings. On rectal palpation, a soft tissue septum (5 cm) with a mucosal surface between the two anal openings was palpated. In addition, circumferential rectal musculature was not appreciated on the ventral aspect. Urogenital evaluation revealed duplication of the vestibule and vagina with a complete centrally located septum extending dorsoventrally. Computed tomography (CT) of the abdomen and pelvis, vaginocystourethrogram, and colonogram were performed. Complete bifurcation of the urinary bladder with duplication of the urethra, cervix, and vaginal canal was noted. Approximately 2 cm from the rectum, there was a similar bifurcation that converged the colon into two rectal portions and separate anal openings. The owner was counseled on the severity of congenital malformations and a high likelihood of aging-related developmental complications in the future. The owner elected humane euthanasia and a necropsy was performed to confirm the malformations.

## 1. Introduction

A diagnosis of complete urogenital and anorectal duplication was made via contrast computed tomography and necropsy in a 10-week-old intact female golden retriever. Clinical signs included smaller, soft, poorly formed, fecal material from both anal openings and urinary incontinence which had been present since birth. Advanced imaging of the patient was pursued, but surgical correction was declined. A necropsy was performed to further investigate the anatomical variations in this patient. These congenital abnormalities were reviewed in the veterinary literature and compared with human descriptions of similar abnormalities.

## 2. Case Description

A 10-week-old 9.6 kg sexually intact, female, golden retriever puppy was purposefully bred and born via cesarean section with ten other littermates (seven females and three males). The female dog in question had two smaller than anatomically normal slit-like anal openings with haired skin coursing between the two distinct orifices. The referring veterinarian examined and explored the female puppy's abnormality after birth and ruled out a persistent anal membrane. No further treatment or surgery was performed since the female puppy did not appear to have difficulty with defecation. The dog was then referred to the University of Florida Small Animal Hospital for further evaluation and exploration of surgical treatment options.

**Figure 1:**
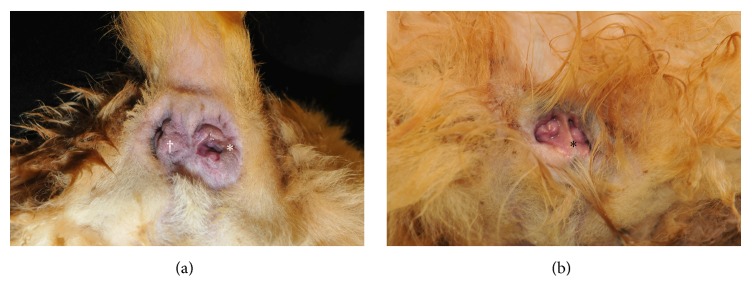
(a) and (b) Two functional anal openings. This photograph depicts the locations of the two anal openings, left (†) and right (*∗*). Both openings were situated off-center from midline by a few millimeters. The left anal opening is positioned more off-center, anatomically narrower, and angled more laterally than the larger right anal opening. (b) Vulva and vestibule. This photograph depicts the complete septum (*∗*) and the small clitoris with smaller than usual openings into each vaginal canal. Dorsal is toward the bottom of the image.

On presentation, the dog was bright, alert, and in good condition, with a body condition score of 5/9 and normal vital signs. The owner reported the patient defecated through both anal openings without tenesmus and noted that the fecal material was mostly soft and of poorly formed consistency. During the physical examination, the dog became excited and dribbled urine in several small spots. Further discussion with the owner revealed that she frequently found urine on the floor but did not recognize this as a sign of incontinence. On physical examination, the left anal orifice was situated 3-4 mm off midline, was smaller in diameter, and had a slit-like appearance compared to the right anal opening, which was 1-2 mm to the right of midline (Figure 1(a)). On rectal digital palpation of both orifices, mild discomfort was initially noted, and a pain response was observed when palpated more cranially. Additionally, a soft tissue septum with a palpable mucosal surface between the two communicating anal openings was found. This surface and septum was palpated and extended approximately 5 cm orally in the colon. In addition, a lack of palpable circumferential rectal musculature was appreciated on the ventral aspect suggesting this dog had an incomplete external anal sphincter. Urogenital physical evaluation revealed a complete ventral to dorsal soft tissue septum bisecting the vulva, which can be seen in Figure 1(b).

Abdominal radiography was obtained to rule out possible orthopedic and vertebral anomalies and to examine the abdominal contents. Abdominal radiography revealed a normal sized stomach containing a small amount of gas and soft tissue material. The small intestines were normal in diameter and had a normal distribution within the abdomen, and a few segments had a small amount of central contiguous gas. The colon was normal in size and contained a small amount of fecal material and gas that extended caudally within the rectum to the perineal margin. Serosal detail was normal with no other abdominal abnormalities noted. No orthopedic or vertebrae malformations were noted. The visible portion of the thorax was within normal limits. Based on these findings, contrast computed tomography (CTA) was recommended to further define the congenital anomalies.

A packed cell volume: 32% (reference range 37-54%), total protein: 4.6 g/dL (reference range 5.4-7.1 g/dL), and AZOstick (within normal limits at 5-15 mg/ml) were performed prior to induction of anesthesia. Premedication and induction of general anesthesia were performed with 1 mg of butorphanol intravenously (0.1 mg/kg), and 30 mg of Propofol (3.13 mg/kg) plus 15 mg ketamine (1.56 mg/kg) respectively and the patient was maintained on isoflurane inhalant ranging from 1 to 2% and oxygen at 1L/min for the duration of the CTA imaging series. Heart rate, electrocardiogram, indirect oscillometric blood pressure, oxygen saturation (by means of pulse oximetry), and end-tidal CO_2_ were monitored throughout the procedure. Lidocaine was administered to the patient during the procedure at 1.66 mg/kg intravenously.

Advanced imaging, by computed tomography (Toshiba Aquilon 8 CT Scanner, Toshiba Medical Systems, Tustin, Calif.) of the abdomen and pelvis, was performed with intravenous administration of a nonionic, iodinated contrast medium, Iohexol (300mg I/ml), for a total of 4.8 g (500 mg/kg). Images were obtained after contrast administration during both arterial and venous phases. Helical CT images were obtained in a volume data set and were reconstructed in soft tissue, bone, and lung algorithms and then reformatted in transverse, dorsal, and sagittal planes.

A vaginocystourethrogram was performed using two 10 French Foley catheters (one in each vulva), using approximately 15 mL of nonionic, iodinated contrast medium (Iohexol 300 mg I/ml) in each catheter to confirm complete or incomplete duplication as well as establish whether communication was present. Subsequently, a retrograde colonogram was performed using two 10 French red rubber catheters (one in each anal orifice), secured by purse string sutures of 2-0 Nylon (Ethilon®) suture. Approximately 30 mL of barium sulfate paste positive contrast medium was administered in each red rubber catheter. The dog was rescanned, and images were acquired after vaginocystourethrogram and retrograde colonogram.

**Figure 2:**
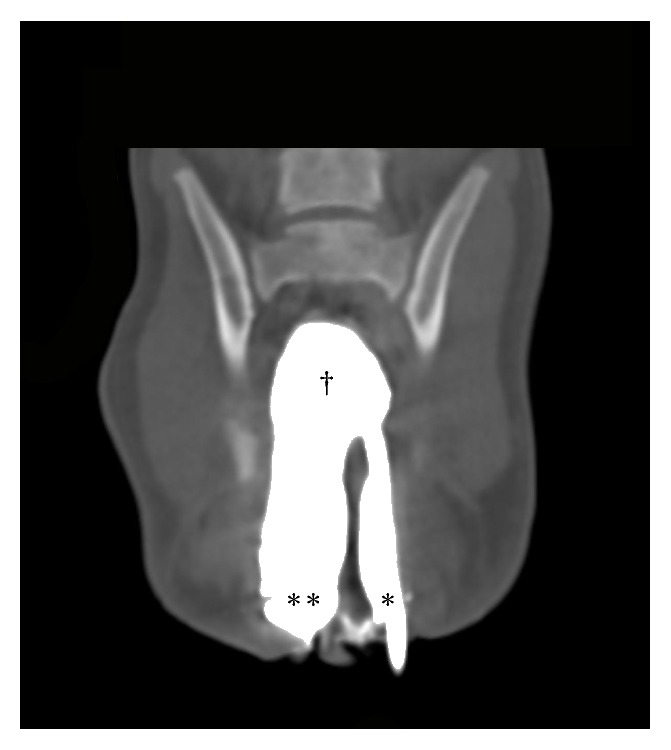
Dorsal plane CT image of the rectum with positive contrast medium infused through two catheters in each anal opening left (*∗∗*) and right (*∗*). The contrast is distinctly separate caudally, proceeding cranially in the rectum, and then joins approximately 4.5 cm cranially (†).

**Figure 3:**
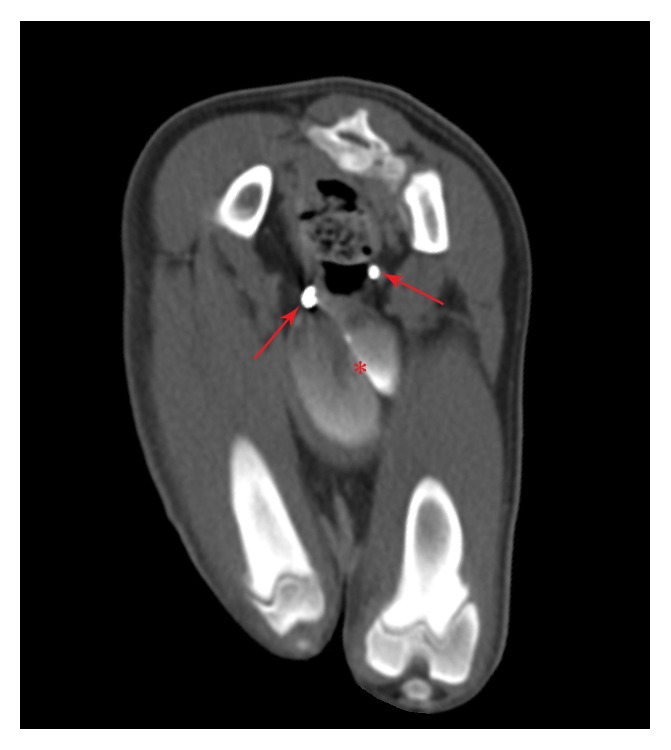
Transverse CT image at the level of the ischium with nonionic, iodinated contrast medium demonstrating a septate urinary bladder (*∗*) and two distinct urethras (arrows).

CT images and abdominal radiographs were reviewed and interpreted by a board-certified radiologist. A vertically oriented soft tissue septum was present and measured 0.37 cm thick and 4.8 cm in length, which extended from the anus to the caudal rectum diverging the positive contrast medium laterally. The contrast then joined again cranially at the level of the second caudal vertebra (Figure 2). The vulva was divided in the sagittal direction by a soft tissue septum that measured 0.38 cm in thickness. The vestibule, urethra, and urinary bladder were duplicated and positioned side-by-side. The urethra was seen coursing ventral to the uterine horns and had minimal contrast medium filling after urethrogram. The left and right portions of the urinary bladder were mildly to moderately filled with fluid and contrast medium (Figure 3). The kidneys and ureters were bilaterally symmetric and within normal limits. The left ureter entered the ureterovesicular junction of the left urinary bladder, and the right ureter entered the ureterovesicular junction of the right urinary bladder, in the region of the trigone at the level of the first caudal vertebrae. The ovaries were present and in a normal anatomic location, immediately caudolateral to their respective kidney. The caudal mesenteric artery was completely absent, and no vessel was observed branching from the aorta between the deep circumflex iliac arteries and the external iliac arteries. In addition to this vasculature anomaly, the cranial mesenteric artery took an aberrant path, coursing caudally, in a left lateral direction immediately to the left of the median sacral artery rather than the typical right lateral direction. The cranial mesenteric artery traveled dorsally to the descending colon, then bifurcated at the level of the L7 vertebra, giving off a cranial rectal artery branch, instead of this vessel normally originating from the caudal mesenteric artery. Orthopedically, there were no abnormalities noted.

Surgical options for correction of the anal abnormalities were presented to the owner. However, due to the urogenital findings and concurrent urinary incontinence, the owner instead elected humane euthanasia. The dog was euthanized with 3 ml of sodium pentobarbital (390mg/ml) given intravenously at 122 mg/kg.

**Figure 4:**
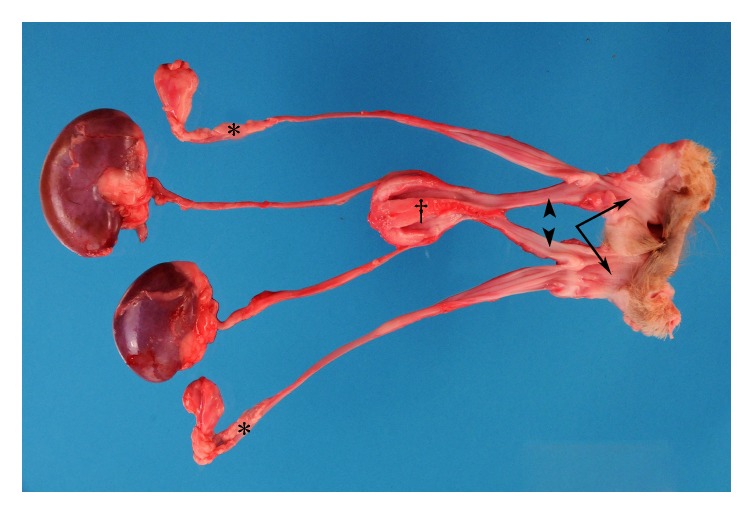
Urogenital system. The dog had normal kidneys and ureters. One ovary and uterine horn on each side were visualized (*∗*). The urinary bladder complete septum (†) was seen. A single urethra (arrowheads) exiting on each side of the urinary bladder and then joining each cervix before exiting through separate vaginal canals (arrows).

**Figure 5:**
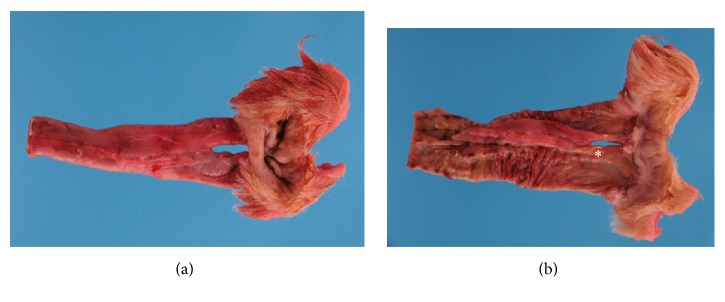
Colon and rectum. (a) Serosal surface and external anal orifices. (b) Mucosal surface and external anal orifices. The mucosal surface of the colon was completely duplicated with a septum (*∗*) which extended 4.5 cm orally before joining to one colon.

A complete necropsy was performed by a board-certified pathologist (MJD). The urinary bladder was abnormal when viewed externally with a cranial-to-caudal oriented central depression. Internally, the urinary bladder was completely bifurcated by a soft tissue septum extending from the apex to the trigone. The trigone had separate urethras exiting in each urinary bladder chamber. The urethras each extended approximately 4.5 cm caudally and then entered separate duplicated vaginas, each with a separate vaginal canal and opening to the outside of the body (Figure 4). The uterine horns each were separate, with each connected to a single ovary and having a separate cervix. The urethras joined the uterine horns at the level of the cervices. The vaginal canals exited caudally through distinct vestibules as seen from the exterior. Approximately 2 cm from the rectum, there was a similar bifurcation that diverged the colon into two rectal portions and separate anal openings (Figure 5). The external anal sphincters of both orifices were thin lacking normal muscular layers and incomplete circumferentially. No significant lesions were detected in the remainder of the organs. The necropsy diagnosis was urinary bladder bifurcation with vaginal and colonic duplication.

## 3. Discussion

This case represents a unique group of anatomic anomalies not previously reported in the dog. Reports in the veterinary literature are limited, encompassing only four canine cases [1–4] and one feline case [5] with congenital colonic or urogenital abnormalities. There are limited surgical treatment options for these anomalies in animals; humane euthanasia is often advocated and elected.

Of the four canine cases, three were not treated surgically. A seven-week-old mixed breed puppy was found to have partial sacral duplication, hemivertebra, and duplication of the bladder, descending colon, and rectum [2]. A nine-week-old male Labrador was presented for ataxia and forelimb lameness, with duplication of the entire colon and malformation of the fourth (T4) and fifth thoracic (T5) vertebral bodies observed at necropsy [4]. A 12-week-old Münsterländer hunting dog with a history of rectal prolapse was diagnosed via laparotomy with a colonic duplication and cecal malformation [3]. Surgical correction was not pursued and the dog was humanely euthanized.

The only reported case of colonic duplication surgically repaired in veterinary medicine was a six-month-old intact female, Boston terrier [1]. This dog did not have any other skeletal or urogenital abnormalities identified, so surgical correction was attainable. Surgical correction consisted of incision into the colon halfway between the mesenteric and antimesenteric borders. An ostium was identified consisting of just mucosa, which was incised and extended orally to within one centimeter of the proximal extent of the duplication. The colon incision was then closed with 3-0 polydioxanone suture in a simple continuous pattern in two layers. Nine months after correction, the patient had no tenesmus or constipation following surgery [1].

There is one report of urinary bladder duplication in a cat with urine soaked perineum and was thought to have ectopic ureters antemortem. The abnormalities were diagnosed at necropsy after presenting dead on arrival 35 months after ovariohysterectomy [5].

To the authors' knowledge, this case represents the most extensive colonic and urogenital malformations reported in a dog in veterinary medicine. There are numerous reports in the human literature describing rectal or colonic duplications [6–14]. Most of these malformations are descriptions in the form of a case series or case report and are usually complex congenital anomalies involving the colon and often the urinary and genital tracts [9].

Duplication of the colon can be associated with complete duplication of the urinary bladder and the urethra, which is a rare anomaly typically reported in children within the first few months of life [10, 15]. Embryologically, there are three discussed mechanisms for colonic duplication reported in the literature, including persistence of the vacuoles present among the masses of epithelial cells during the solid stage of intestinal development, partial or incipient twinning of the primitive colon and rectum after division of the cloaca by the urorectal folds [2] and splitting of the notochord during embryological development [13]. Anorectal malformations are thought to result from abnormal development of the urorectal septum in prenatal life and have been characterized and classified by the Krickenbeck Conference of 2005 [9]. Despite multiple theories, no conclusive evidence explains the constellation of the abnormalities described in people and this case.

In the human literature, Kottra and Dodds reported a classification, consisting of two types, for colonic duplication based on the work of Smith [12, 16]. A type I colonic duplication involves spherical, tubular, double-barreled, loop, or multiple duplications. A type II colonic duplication is usually a double-barreled duplication with duplications of the urinary or genital tracts. With type II duplication, interestingly, the anal openings usually lie on either side of midline and is associated with double genitals, double urethras, or bladders as seen in our patient [12]. Interestingly, the rectum is the least commonly reported alimentary tract duplication, with small intestinal duplication being the most common [17].

Urinary or urogenital malformations in humans come in two forms: those patients with clinical signs that present early in life, and others that remain asymptomatic for years [18]. Human patients are surgically corrected to preserve quality of life and to correct obvious physical deformities that may result in esteem concerns later in life. Interestingly, duplication of the urethra was found to have a male preponderance in individuals ranging from 4 months to 10 years of age on presentation with anal urine voiding and incontinence as presenting symptoms [19]. Reports in another study describe duplication of the external genitalia or of the lower intestinal tract, comparable to the female dog described in our case, as the most common [7].

In addition to the colonic and urogenital malformations, the dog described in this case report also lacked a caudal mesenteric artery. The caudal mesenteric artery provides blood supply to the distal colon and rectum. Variations of the caudal mesenteric artery are previously described in dogs. In a recent study of five normal dogs, the caudal mesenteric artery branched off the aorta in a leftward direction in three dogs (60%) and in a rightward direction in two dogs (40%) [20].

In our patient, the urogenital and colonic malformations were detected early in life. Both surgical and medical treatment options were discussed with the owner. Surgical repair options were limited and carried a high risk of morbidity without potential benefit to the patient. The owner raised concerns about the dog's quality of life and concern for multiple surgical procedures, so humane euthanasia of the dog was elected. This case represents the intricate process of embryonic development, the critical utilization of cross-sectional imaging with regard to surgical planning, and the utility of correlating cross-sectional imaging which verify the anatomic pathology findings.
